# Hospital burden of influenza, respiratory syncytial virus, and other respiratory viruses in Canada, seasons 2010/2011 to 2018/2019

**DOI:** 10.17269/s41997-025-01049-x

**Published:** 2025-07-07

**Authors:** Abbas Rahal, Andrea Nwosu, Dena L. Schanzer, Christina Bancej, Amanda Shane, Liza Lee

**Affiliations:** Centre for Emerging and Respiratory Infections and Pandemic Preparedness, Public Health Agency of Canada, Ottawa, ON Canada

**Keywords:** Influenza, Respiratory syncytial virus, Surveillance, Disease burden, Respiratory infectious diseases, Grippe, Virus respiratoire syncytial, Surveillance, Charge de morbidité, Maladie respiratoire infectieuse

## Abstract

**Objectives:**

The objective of this study was to develop a model to estimate the hospitalization burden attributable to influenza, respiratory syncytial virus (RSV), enterovirus (EV), human metapneumovirus (HMPV), human parainfluenza virus (HPIV), and other respiratory viruses (OV) in Canada.

**Methods:**

A Poisson regression model was developed using respiratory hospitalization administrative data for the seasons 2010/2011 to 2018/2019.

**Results:**

The estimated average seasonal number of respiratory hospitalizations attributable to influenza was 15,000 in Canada (rate 43.4 hospitalizations per 100,000 population [95%CI 40.9, 46.0]), and 13,000 (rate 36.3 hospitalizations per 100,000 population [95%CI 29.2, 43.4]) for RSV. The estimated average seasonal numbers of hospitalizations attributable to EV, HMPV, HPIV, and OV were 6000 (rate 16.2 hospitalizations per 100,000 population [95%CI 10.7, 21.8]), 4000 (rate 12.4 hospitalizations per 100,000 population [95%CI 7.1, 17.6]), 2000 (rate 5.9 hospitalizations per 100,000 population [95%CI 2.0, 9.8]), and 3000 (rate 8.9 hospitalizations per 100,000 population [95%CI 0.04, 17.7]), respectively.

**Conclusion:**

This study provided updated and new Canadian estimates for hospitalizations attributable to influenza, RSV, EV, HMPV, and HPIV for 2010/2011 to 2018/2019 surveillance seasons. These estimates are important given the emergence of SARS-CoV-2 and the ongoing circulation of seasonal respiratory viruses. Routine burden estimation is pivotal in supporting the implementation and evaluation of public health programs focused at mitigating the impacts of respiratory viruses. Although multiple external factors are at play, this study indicates that influenza and RSV attributable hospitalizations were persisting and generally increasing in Canada in recent years preceding the COVID-19 pandemic.

## Résumé

**Objectifs:**

L’objectif de cette étude était de développer un modèle pour estimer le fardeau des hospitalisations attribuable à la grippe, au virus respiratoire syncytial (VRS), à l’entérovirus (EV), au métapneumovirus humain (MPVH), au virus parainfluenza humain (VPIH) et à d’autres virus respiratoires au Canada.

**Méthodes:**

Un modèle de régression de Poisson a été développé en utilisant des données administratives sur les hospitalisations pour causes respiratoires pour les saisons 2010–2011 à 2018–2019.

**Résultats:**

Le nombre moyen estimé d’hospitalisations saisonnières pour causes respiratoires attribuables à la grippe était de 15 000 au Canada (taux de 43,4 hospitalisations pour 100 000 habitants [intervalle de confiance (IC) à 95%: 40,9–46,0]), et 13 000 (taux de 36,3 hospitalisations pour 100 000 habitants [IC à 95%: 29,2–43,4]) pour le VRS. Le nombre moyen estimé d’hospitalisations saisonnières attribuables à l’EV, au MPVH, au VPIH et à d’autres virus respiratoires était de 6 000 (taux de 16,2 hospitalisations pour 100,000 habitants [IC à 95%: 10,7–21,8]), 4 000 (taux de 12,4 hospitalisations pour 100 000 habitants [IC à 95%: 7,1–17,6]), 2 000 (taux de 5,9 hospitalisations pour 100 000 habitants [IC à 95%: 2,0–9,8]), et 3 000 (taux de 8,9 hospitalisations pour 100 000 habitants [95% IC: 0,04–17,7]), respectivement.

**Conclusion:**

Cette étude a fourni des estimations canadiennes mises à jour et nouvelles pour les hospitalisations attribuables à la grippe, au VRS, à l’EV, au MPVH et au VPIH pour les saisons de surveillance de 2010–2011 à 2018–2019. Ces estimations sont importantes compte tenu de l’émergence du SRAS-CoV-2 et de la circulation continue des virus respiratoires saisonniers. L’estimation de la charge routinière est essentielle pour soutenir la mise en œuvre et l’évaluation des programmes de santé publique axés sur l’atténuation des impacts des virus respiratoires. Bien que plusieurs facteurs externes soient en jeu, cette étude indique que les hospitalisations attribuables à la grippe et au VRS persistaient et augmentaient généralement au Canada au cours des dernières années précédant la pandémie de COVID-19.

## Introduction

Globally, respiratory viruses such as influenza and respiratory syncytial virus (RSV) result in substantial morbidity and mortality, especially among young children and older adults above the age of 65 (Iuliano et al., 2018; Li et al., 2020; Nair et al., 2013; Paget et al., 2019; Shi et al., 2019; Troeger et al., 2019). In Canada, respiratory virus disease epidemics occur each year typically starting in late fall and ending in late spring (National Advisory Committee on Immunization, 2023). As in other countries globally, these disease epidemics have been associated with increased hospitalizations and deaths and strain on the healthcare system (Fleming & Elliot, 2005; Schanzer et al., 2012, 2013, 2017).

The Public Health Agency of Canada (PHAC) monitors the spread of respiratory viruses through its national respiratory virus surveillance system (Public Health Agency of Canada, 2025). Although this surveillance system is able to provide timely characterization of respiratory virus activity, patterns, and trends, it is unable to provide a measure of the full burden of illness associated with these viruses due to many factors, including underreporting, under-ascertainment, limited geographic and population coverage, societal factors, and epidemiological complexities of disease presentation. Furthermore, national administrative hospital databases that capture respiratory viral hospitalizations are also subject to underreporting and under-ascertainment as laboratory testing for seasonal respiratory viruses is not performed on every individual with respiratory symptoms and may produce false negative results (Schanzer et al., 2013, 2017). Due to these limitations in both surveillance and administrative datasets, the World Health Organization (WHO) advises member countries to use burden of disease studies to estimate the impact of respiratory viruses on the population (World Health Organization, 2015).

Quantifying the burden of respiratory viral illness is crucial for determining the risk of morbidity and mortality in the population, evaluating public health programs, informing vaccination guidance, allocating resources, and mitigating the impacts of these viruses on the population. Many countries have conducted influenza burden of disease studies and have incorporated this work into their surveillance programs (World Health Organization, 2015). There are various methods for estimating the morbidity and mortality of respiratory viral infections, and the choice of method depends on the availability of data sources, the quality of the data in terms of representativeness, accuracy, completeness, and ability to account for associated biases (World Health Organization, 2015). In Canada, most respiratory virus disease burden studies rely on hospital administrative discharge records and Poisson regression models to derive estimates due to the high quality of the data source and lack of systematic testing of individuals for respiratory viruses (Schanzer et al., 2013, 2017). Hospital administrative data are available for multiple years, the most representative of the Canadian population (covering 77%) among available data sources, and include age-specific information.

Since the most recent Canadian estimates of the hospital burden associated with respiratory virus infections were published in 2017, this study aimed to achieve three key objectives (Schanzer et al., 2017). First, provide updated estimates of season-specific hospitalization burden attributable to influenza and RSV. Second, provide new burden estimates for other seasonal respiratory viruses (enterovirus, human metapneumovirus, human parainfluenza virus, and other seasonal respiratory viruses) in Canada during the period between the 2009 H1N1 pandemic and the COVID-19 pandemic (seasons 2010/2011 to 2018/2019). Finally, develop a reference statistical model for estimating respiratory virus burden so that routine annual burden estimation for seasonal respiratory viruses can be incorporated into Canada’s national respiratory virus surveillance program.

## Methods

### Design

PHAC uses a statistical model-based approach to produce national hospitalization burden estimates for influenza, RSV, and other respiratory viruses. This model has been used in the past to produce influenza burden estimates both nationally and internationally and is one of the methods suggested by WHO in their practical guide for designing and conducting influenza disease burden studies (Schanzer et al., 2012, 2013, 2017; Thompson et al., 2003, 2004; World Health Organization, 2015; Wong et al., 2006).

The overall model design remains similar to its predecessor; however, data and some independent variables have been modified (Schanzer et al., 2017). The statistical model estimates the excess morbidity attributable to the circulation of specific respiratory viruses. Excess morbidity is defined as the number of additional hospitalizations that is above the expected number of hospitalizations that would have occurred if the virus had not circulated (Schanzer et al., 2012). The model is based on the assumption that hospitalizations due to non-respiratory illnesses remain consistent over time (i.e., baseline hospitalizations) and that epidemics of respiratory viruses increase the number of hospitalizations above this baseline in proportion to the level of respiratory viral activity (Schanzer et al., 2012). These excess hospitalizations are directly and indirectly caused by respiratory virus circulation and can be used to estimate the impact of the virus on the population and the healthcare system (Schanzer et al., 2012).

### Data sources

#### Canadian viral hospitalization data

Hospital discharge records for all patients admitted to an acute care hospital for a respiratory condition between September 2010 and August 2019 were extracted from the Canadian Institute of Health Information (CIHI) Discharge Abstract Database (DAD) (Canadian Institute for Health Information, 2024). The DAD is a hospital administrative database that contains demographic, administrative, and clinical data on all discharges from acute inpatient facilities in all Canadian provinces and territories, except Quebec, representing 77% of the Canadian population (Canadian Institute for Health Information, 2024). The International Classification of Diseases and Related Health Problems, Tenth Revision, Canada (ICD-10-CA) was used for coding diagnoses (Canadian Institute for Health Information, 2006) over the study period. A hospitalization due to a primary respiratory condition was defined as any record with an ICD-10-CA J code (i.e., diseases of the respiratory system J00-J99) in diagnostic code 1 (Canadian Institute for Health Information, 2006).The DAD allows up to 25 diagnostic codes to be used to describe a hospital stay. Hospital admissions coded with J09 or J10 found anywhere from diagnosis 1 through 25 were used as a proxy for influenza activity. While clinical diagnoses of influenza-like illness (ILI) have been used as a measure of influenza activity, ILI (J11) is not specific to influenza and was not included in this study. Admissions coded with J12.1, J20.5, J21.0, or B97.4 found anywhere from diagnosis 1 through 25 were used as a proxy for RSV activity. We included other proxy variables for enterovirus (EV), human metapneumovirus (HMPV), human parainfluenza virus (HPIV), and other respiratory viruses (OV) in our model. OV is an aggregate variable that included adenovirus, human coronavirus, rhinovirus, and other viral pneumonia. Details of codes used for these proxy variables are presented in Table 1.

**Table 1:** Dependent and independent variables reflecting viral activity, seasonality, and trend in Canada

Independent and dependent variables	Definition
NAdms (dependent variable)	Weekly number of primary respiratory hospitalizations admissions. The week starts on Sunday and ends on Saturday. Weekly number of hospital records with ICD-10 CA J code (i.e., disease of the respiratory system J00-J99) in diagnostic code 1.
Fluproxy	Proxy measure for weekly influenza activity derived based on influenza hospital discharge records. Where ${\beta }_{8,y}$ ($y\; \text {is the value for each season}$) is its corresponding parameter to be estimated by the model. Weekly number of hospital records with ICD-10-CA code of J09 or J10 in any diagnostic code position (diagnoses 1 to 25).
RSVproxy	Proxy measure for weekly RSV activity derived based on RSV hospital discharge records. Where ${\beta }_{9}$ is its corresponding parameter to be estimated by the model. Weekly number of hospital records with ICD-10-CA code of J12.1, J20.5, J21.0, or B97.4 in any diagnostic code position (diagnoses 1 to 25).
EVproxy	Proxy measure for weekly EV activity derived based on EV hospital discharge records. Where ${\beta }_{10}$ is its corresponding parameter to be estimated by the model. Weekly number of hospital records with ICD-10-CA code of J20.3, J20.7, B34.1, or B97.1 in any diagnostic code position (diagnoses 1 to 25).
HMPVproxy	Proxy measure for weekly HMPV activity derived based on HMPV hospital discharge records. Where ${\beta }_{11}$ is its corresponding parameter to be estimated by the model. Weekly number of hospital records with ICD-10-CA code of J12.3 or J21.1 in any diagnostic code position (diagnoses 1 to 25).
HPIVproxy	Proxy measure for weekly HPIV activity derived based on HPIV hospital discharge records. Where ${\beta }_{12}$ is its corresponding parameter to be estimated by the model. Weekly number of hospital records with ICD-10-CA code of J12.2 or J20.4 in any diagnostic code position (diagnoses 1 to 25).
OVproxy	Proxy measure for weekly OV activity based on OV hospital discharge records. Where ${\beta }_{13}$ is its corresponding parameter to be estimated by the model. Weekly number of hospital records with ICD-10-CA code of B34.0, B97.0, J12.0, B34.2, B97.2, J12.8, or J20.6 in any diagnostic code position (diagnoses 1 to 25).
Mon_m_	Categorical variable that accounts for the month in which the admissions occurred. *m* varies between 1 and 13 (instead of 12) because September was divided into two equal parts (9.1 and 9.2) to account for the effect of each part on hospital admissions^¥^. Where ${\beta }_{1,m}$ is its corresponding parameter to be estimated by the model. This variable is used to account for monthly patterns in respiratory hospital admissions.
Cos (*t*) Sin (t) *t* = 2π*week/52.177457	Sinusoidal terms to account for seasonal variation in the weekly number of respiratory hospital admissions. Where *week* is week sequentially numbered. Where ${\beta }_{2}$ and ${\beta }_{3}$ are respectively the parameters of Cos (*t*) and Sin (*t*) to be estimated by the model.
Holiday	Number of statutory holidays in a week. This variable accounts for potential effects that holidays have on respiratory hospital admissions (i.e., admissions are generally lower during the holiday periods). Where ${\beta }_{5}$ is its corresponding parameter to be estimated by the model.
Dec25	Number of weekdays (Monday to Friday) between December 27 and 31. This variable accounts for the potential effect on hospital admissions after Christmas holidays. Where ${\beta }_{6}$ is its corresponding parameter to be estimated by the model.
Jan1	Indicator variable for the first week of January. This variable accounts for the potential effect on hospital admissions after New Year holidays. Where ${\beta }_{7}$ is its corresponding parameter to be estimated by the model.
FY_y_	Categorical variable that accounts for the surveillance season beginning in September and ending in August in which the admissions occurred. Where ${\beta }_{4,y}(y\; \text{is the value for each season})$ is its corresponding parameters to be estimated by the model.
*ε*	Represents the error term.

^¥^The parameter estimate of September was not statistically significant. To make it statistically significant, it was split into two parts based on the number of hospitalizations observed. The number of hospitalizations is higher in the second half of September (in Canada) than in the first half

### Population census data

Canadian population data were obtained from census and intercensal projections published by Statistics Canada (Statistics Canada, 2024).

### Statistical analysis

#### Regression model

**Fig. 1:**
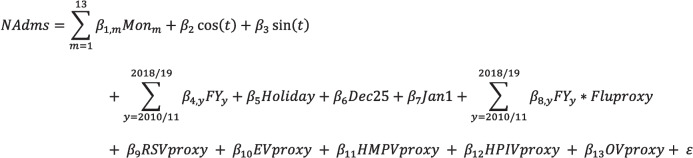
Poisson regression model for estimating the hospitalization burden of influenza, RSV, and other respiratory viruses

A quasi-Poisson regression model was used to determine the share of excess respiratory hospital admissions attributable to influenza, RSV, EV, HMPV, HPIV, and OV circulation. This model is similar to models used in previous publications that estimate the influenza burden in Canada (Schanzer et al., 2012, 2017). The weekly number of primary respiratory hospitalizations was modeled as a function of viral activity, seasonality, and trend. Details of the model are outlined in Fig. 1 and variable definitions are described in Table 1. Burden estimates were reported as both counts and rates. All analyses were performed using SAS PC 9.4 for Windows with the PROC GENMOD procedure by specifying Poisson distribution, the identity link function, expected Fisher information matrix, two-sided confidence intervals, and a dispersion parameter.

### Calculation of hospitalization rates

The statistical model generates estimates of hospitalizations attributable to each virus for Canada, excluding the province of Quebec, as Quebec does not participate in the DAD. We assumed that Quebec’s respiratory virus hospitalization rate per 100,000 population is equal to the estimate for the rest of Canada.

To compute the seasonal rates of hospitalizations per 100,000 population attributable to influenza and RSV in Table 4, we used the outcomes of the statistical model and the population corresponding to start of each surveillance season (i.e., for surveillance season 2010/2011, the annual population estimate for 2010 was used to calculate the rate). Similarly, we used the average seasonal number of hospitalizations attributable to influenza, RSV, EV, HMPV, HPIV, and OV generated by the model and the average population of Canada for the period 2010–2018 to calculate the rates in Table 3.

## Results

During the surveillance seasons from 2010/2011 to 2018/2019 (beginning in September and ending the following August), there were an average of 189,579 primary respiratory hospitalization records in the DAD database per season (Table 2). For the whole study period, there was a total of 1,706,215 primary respiratory hospitalization records which represented 7.5% of the total number of hospitalizations (22,812,400). Overall, influenza and RSV were identified in 3.2% and 3.0% of recorded primary respiratory hospitalizations, respectively. In comparison, EV, HMPV, HPIV, and OV were identified in 0.4%, 0.2%, 0.1%, and 0.5% respiratory hospitalizations, respectively.

**Table 2:** Number and proportion of primary respiratory hospital admission records with respiratory viruses identified, Discharge Abstract Database (DAD), Canada excluding Quebec, 2010/2011 to 2018/2019

Season	Primary respiratory hospitalizations	Influenza identified		RSV identified		EV identified		HMPV identified		HPIV identified		OV identified	
		Number of DAD hospitalizations	%*	Number of DAD hospitalizations	%*	Number of DAD hospitalizations	%*	Number of DAD hospitalizations	%*	Number of DAD hospitalizations	%*	Number of DAD hospitalizations	%*
2010/2011	177,060	2178	1.2	4775	2.7	255	0.1	215	0.1	152	0.1	405	0.2
2011/2012	173,006	1245	0.7	4584	2.6	249	0.1	270	0.2	116	0.1	413	0.2
2012/2013	185,792	4009	2.2	5645	3.0	239	0.1	258	0.1	139	0.1	457	0.2
2013/2014	182,547	4542	2.5	4283	2.3	489	0.3	372	0.2	183	0.1	754	0.4
2014/2015	197,014	6819	3.5	5888	3.0	1043	0.5	301	0.2	237	0.1	976	0.5
2015/2016	188,320	5908	3.1	4659	2.5	823	0.4	443	0.2	228	0.1	1017	0.5
2016/2017	199,840	7005	3.5	7029	3.5	1155	0.6	478	0.2	317	0.2	1191	0.6
2017/2018	203,599	12,260	6.0	6261	3.1	1232	0.6	783	0.4	291	0.1	1264	0.6
2018/2019	199,037	10,477	5.3	7441	3.7	1226	0.6	651	0.3	417	0.2	1254	0.6
Total	1,706,215	54,443	3.2	50,565	3.0	6711	0.4	3771	0.2	2080	0.1	7731	0.5
Average seasonal	189,579	6049	3.2	5618	3.0	746	0.4	419	0.2	231	0.1	859	0.5

*Values are rounded to one decimal place

**Fig. 2:**
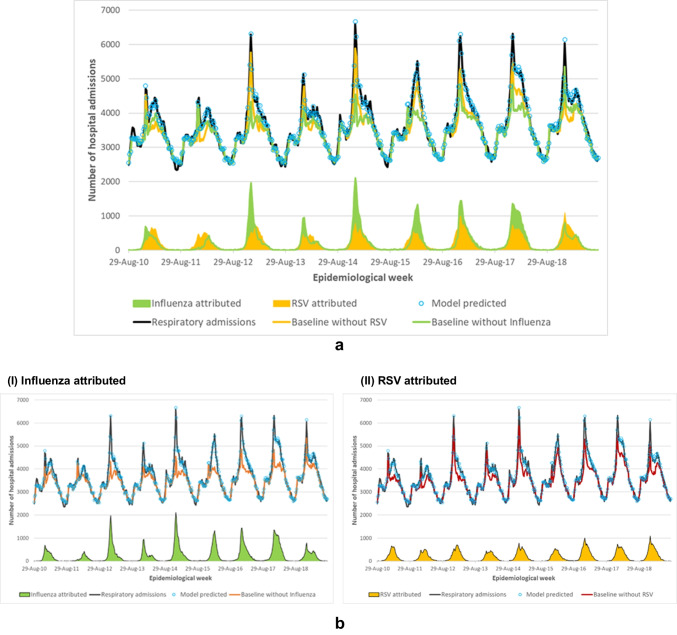
**a** Weekly number of respiratory hospital admissions, model predictions, baseline estimates*, and influenza and RSV attribution (seasons 2010/2011–2018/2019), excluding Quebec, Canada. **b** Weekly number of respiratory hospital admissions, model predictions, baseline estimates*, by (I) influenza and (II) RSV attribution (seasons 2010/2011–2018/2019), excluding Quebec, Canada. *The baseline curve represents expected admissions in the hypothetical absence of (I) influenza and (II) RSV. The shaded area between the baseline and predicted curves indicates excess admissions attributable to (I) influenza and (II) RSV

The average seasonal number of hospitalizations attributable to influenza was estimated to be 15,000 per season in Canada (rate 43.4 hospitalizations per 100,000 population [95%CI 40.9, 46.0]) (Table 3). Seasonally, estimates of influenza attributable hospitalizations ranged from 5000 hospitalizations (rate 15.9 hospitalizations per 100,000 population [95%CI 9.7, 22.2]) during the 2011/2012 season to 24,000 hospitalizations (rate 65.6 hospitalizations per 100,000 population [95%CI 56.1, 75.0]) during the 2017/2018 season (Fig. 2a, b; Table 4). For the last five seasons, 2014/2015–2018/2019, the average seasonal number of hospitalizations was estimated at 19,000 per season in Canada, corresponding to an estimated rate of 51.8 hospitalizations per 100,000 population [95%CI 48.0, 55.5].

**Table 3:** Estimated average seasonal number and rate of hospitalizations attributable to influenza, RSV, EV, HMPV, HPIV, and OV, Canada, seasons 2010/2011 to 2018/2019

Virus	# Hospitalizations*	Rate (95% CI) per 100,000 population**
Influenza	15,000	43.4 (40.9, 46.0)
RSV	13,000	36.3 (29.2, 43.4)
EV	6000	16.2 (10.7, 21.8)
HMPV	4000	12.4 (7.1, 17.6)
HPIV	2000	5.9 (2.0, 9.8)
OV***	3000	8.9 (0.04, 17.7)

*Outcomes were prorated to obtain estimates for all of Canada as the statistical model generates the estimates without empirical data from the province of Quebec. Values are rounded to the nearest thousand

**The statistical model generates estimates for Canada, excluding the province of Quebec, as Quebec does not participate in the DAD. Values are rounded to the level of statistical significance

***OV included adenovirus, human coronavirus, rhinovirus, and other viral pneumonia

**Table 4:** Estimates of seasonal number and rate of hospitalizations attributable to influenza and RSV, Canada, 2010/2011 to 2018/2019

Season	Influenza		RSV	
	# Attributed*	Rate per 100,000 population (95% CI)**	# Attributed*	Rate per 100,000 population***
2010/2011	10,000	28.3 (21.3, 35.4)	11,000	32.3
2011/2012	5000	15.9 (9.7, 22.2)	11,000	30.7
2012/2013	19,000	54.5 (48.4, 60.6)	13,000	37.3
2013/2014	11,000	30.5 (24.0, 37.1)	10,000	28.0
2014/2015	23,000	63.5 (56.4, 70.7)	13,000	38.1
2015/2016	15,000	43.2 (37.4, 49.1)	11,000	29.8
2016/2017	19,000	53.3 (45.1, 61.6)	16,000	44.5
2017/2018	24,000	65.6 (56.1, 75.0)	14,000	39.1
2018/2019	12,000	33.7 (23.5, 43.9)	17,000	45.8

*Outcomes were prorated to obtain estimates for all of Canada as the statistical model generates the estimates without empirical data from the province of Quebec. Values are rounded to the nearest thousand

**The statistical model generates estimates for Canada, excluding the province of Quebec, as Quebec does not participate in the DAD. Values are rounded to the level of statistical significance

***Annual rates are calculated based on the model output which is the difference between the predicted number of respiratory admissions and the baseline representing the expected number of respiratory admissions in the hypothetical absence of RSV

The average seasonal number of hospitalizations attributable to RSV was estimated to be 13,000 (rate 36.3 hospitalizations per 100,000 population [95%CI 29.2, 43.4]) per season in Canada (Table 3). Over the study period, the burden attributable to RSV ranged from 10,000 (rate 28.0 hospitalizations per 100,000 population) during the 2013/2014 season to 17,000 (rate 45.8 hospitalizations per 100,000 population) hospitalizations with the highest estimated burden occurring in the 2018/2019 surveillance season. For the last five seasons, 2014/2015–2018/2019, the average seasonal number of hospitalizations attributable to RSV was estimated at 14,000 per season, corresponding to an estimated rate of 39.5 hospitalizations per 100,000 population. Comparing the hospitalization records data to the model-based results, measured hospitalizations underestimated the burden of hospitalization by 49% for influenza and 43% for RSV on average each season.

The average seasonal numbers of hospitalizations per season attributable to EV, HMPV, HPIV, and OV were estimated to be 6000 (rate 16.2 hospitalizations per 100,000 population [95%CI 10.7, 21.8]), 4000 (rate 12.4 hospitalizations per 100,000 population [95%CI 7.1, 17.6]), 2000 (rate 5.9 hospitalizations per 100,000 population [95%CI 2.0, 9.8]), and 3000 (rate 8.9 hospitalizations per 100,000 population [95%CI 0.04, 17.7]), respectively (Table 3). The OV estimate is unstable and shows significant dispersion; this is evident by its wide confidence interval and this differs from influenza and RSV which had tight confidence intervals.

## Discussion

This study produced updated model-based estimates of hospitalization burden attributable to influenza and RSV in the Canadian population for the 2010/2011 to 2018/2019 surveillance seasons. Based on the model, it is estimated that on average for each surveillance season in Canada, 15,000 hospitalizations are attributable to influenza (43.4 hospitalizations per 100,000 population) and 13,000 hospitalizations are attributable to RSV (36.3 hospitalizations per 100,000 population). Estimates varied from season to season: from 5000 to 24,000 hospitalizations (15.9 to 65.6 hospitalizations per 100,000) for influenza and from 10,000 to 17,000 (28.0 to 45.8 hospitalizations per 100,000) for RSV.

The influenza and RSV estimates reported in this study are higher than estimates reported in the previous Canadian study conducted by Schanzer et al. (2017). One reason for the increase in the estimates may be due to an increase in viral activity over the study period. There were 208% more influenza and 20% more RSV hospitalization records during this study period compared to the previous one of Schanzer et al. (2017). One contributing factor to this increase is the definition used for the influenza proxy variable. The previous Schanzer et al. (2017) model used only the ICD-10-CA code J10, whereas the current influenza proxy variable was based on both J09 and J10, with J09 accounting for approximately 22% of the total identified influenza hospitalizations (J09 + J10). Another contributing factor to this increase may be the increase in the percentage of respiratory hospitalization records with a viral identification. Identified influenza increased from 1% to 5% and RSV from 2.7% to 3.4% among respiratory hospitalizations with some variation season to season. These two factors are directly affected by testing practices in Canada. During this study period, there was evidence of increasing influenza and RSV testing (Public Health Agency of Canada, 2024a). Additionally, compared to the study periods of Schanzer et al. (2017), there has been a change in influenza and RSV testing from virus culture to reverse-transcriptase polymerase chain reaction (PCR) testing, a much more sensitive test (Patel et al., 2012; Chartrand et al., 2015). While the change and increase in testing have contributed to increased viral identification and may have contributed to the increase in the burden estimates, it also aids in improving the accuracy of the estimates.

During the study period, primary respiratory hospitalizations were variable season to season but were on a general upward trend. Increased viral testing and identification and characteristics of the circulating viruses (such as dominant type of influenza and RSV and subtype of influenza) play a direct role in the number of influenza and RSV-associated hospitalizations by season and may play a large role in the variation in the number of hospitalizations each season. There are some other epidemiological and ecological factors that may also be contributing to this increase, such as vaccine coverage and effectiveness, population immunity to circulating viruses, and population demographics (i.e., the aging Canadian population, the increasing number of adults with comorbidities such as diabetes that place them at increased risk of influenza complications) (Public Health Agency of Canada, 2024b; Public Health Agency of Canada, 2023; Statistics Canada, 2016, 2024; Goka et al., 2014; Mulpuru et al., 2019). With the emergence and persistence of SARS-CoV-2 and this observed general increase in hospitalizations records identified with influenza or RSV, it is an indication that additional pressures on hospitals in Canada will continue, especially during the winter months.

In addition to updated estimates of influenza and RSV, this study provided, for the first time, national virus-specific estimates for other circulating respiratory viruses that are routinely monitored in Canada. This includes human metapneumovirus (HMPV), enterovirus (EV), and human parainfluenza virus (HPIV). Although previous Canadian studies have produced estimates of hospitalization burden attributable to other circulating viruses, these estimates have been an aggregate measure for all other circulating viruses and not virus-specific (Schanzer et al., 2017). The model estimated that on average, each surveillance season, 6000, 4000, and 2000 hospitalizations are attributable to EV, HMPV, and HPIV (16.2, 12.4, and 5.9 hospitalizations per 100,000 population), respectively. The estimates for these other circulating viruses were 3 to 7 times lower than for influenza and 2 to 6 times lower than for RSV, which is expected considering the testing volumes for viruses are also lower than those for influenza and RSV (Public Health Agency of Canada, 2024a). Although the individual estimated hospitalization rates for HMPV, EV, HPIV, and OV are lower than those of influenza and RSV, collectively as a group of seasonal respiratory viruses, they account for a notable volume of respiratory hospitalizations attributed to a virus in a given season. This provides a strong rationale to continue to monitor the activity of these viruses in addition to influenza, RSV, and SARS-CoV-2.

The tertiary objective of the study was the development of a working model that can be used by Canada’s national respiratory virus surveillance program to produce annual estimates not only for influenza, but also for RSV and other seasonal circulating viruses. PHAC has recently implemented an integrated approach for respiratory virus surveillance. The goal is to unify respiratory virus–specific surveillance systems into a single cohesive framework that maximizes our capacity to sustainably monitor, respond to, and mitigate high-impact respiratory viruses. This model-based multi-virus burden estimation approach aligns with this integrated surveillance approach and is important for capturing the full burden of illness attributable to high-impact respiratory viruses in Canada.

### Limitations

There are numerous statistical models that can be used to estimate the burden of influenza, each with their advantages and disadvantages. The Poisson regression model, and its different forms, is one of the most widely used models to generate burden estimates (Gilca et al., 2009; Thompson et al., 2004; Yang et al., 2011). The availability and quality of data often limit which method can be used within a particular country. While the updated Poisson regression model used in this study still has the same advantages and limitations as described in previous publications by Schanzer et al. (2012, 2017), the model continues to be used as the performance is satisfactory and is compatible with available data in Canada.

Life stage (age-group)–specific (i.e., pediatric, adult, and senior) estimates were not a primary objective of this study. While these estimates are sufficient to provide initial overall respiratory burden estimates in Canada, life stage–specific models that account for differences within influenza, RSV, and other respiratory viruses testing practices need to be developed. A key assumption of the model is that testing for influenza, RSV, and other respiratory viruses is the same across all different life stage groups. In Canada, testing practices for respiratory viruses use risk-based approaches (i.e., prioritize based on risk of influenza complications, severity, outbreak specimens, and other factors) which differ among the pediatric and adult populations. Respiratory diseases also have a higher burden in the youngest and oldest age groups. Life stage–specific estimates would further support the work on informing and evaluating public health policies and programs targeting specific age groups and would potentially provide more accurate estimates of respiratory viral burden in Canada.

The wide confidence interval of rate estimates for other seasonal respiratory viruses is likely the result of poor statistical power due to relatively low activity and/or consistent year-round circulation without a clear epidemic wave. Adenovirus, human coronavirus, rhinovirus, and other viral pneumonia model outputs were not statistically significant and were combined as OV, which produced a statistically significant estimate but a wide confidence interval. While the point estimates are not precise, the confidence intervals are useful in indicating the potential burden associated with these viruses.

Finally, due to unavailability of Quebec data in the DAD, we assumed that the rates of respiratory hospitalizations attributable to influenza, RSV, and other respiratory viruses of Quebec are equal to those of the rest of Canada, which is similar to what was done in the past (Schanzer et al., 2012, 2013, 2017). This assumption may have an impact on the estimated number of respiratory hospitalizations for Canada; therefore, to improve our estimates, the feasibility to gain access to Quebec DAD data for inclusion in our future modelling work will be explored.

## Conclusion

Burden of disease studies are important for estimating the impact of respiratory viruses on the population. In Canada, the influenza and RSV estimates reported in this study demonstrate the importance of incorporating burden estimation into routine surveillance work in order to understand the impact of these viruses on the Canadian population and healthcare system. The regression results demonstrate that the administrative hospitalization database, which is considered our most complete source for hospitalization data in Canada, under-represents the burden associated with seasonal influenza and RSV by 49% and 43%, respectively. With new therapeutics like RSV vaccines, evolving respiratory virus patterns, demographic changes, and the potential emergence of new viruses of epidemic potential, incorporating routine burden estimation into our existing surveillance program is essential. These estimates will help inform and evaluate public health policies, programs, and communications that are implemented to mitigate the impacts of respiratory viruses on the Canadian population. With the establishment of this reference model, immediate future work will involve using this working model to develop age-group–specific estimates to address differences in respiratory virus testing and hospitalizations for children and adults, modifying or incorporating variables to account for the activity of SARS-CoV-2 and develop burden estimates for SARS-CoV-2.

## Contributions to knowledge

What does this study add to existing knowledge?

- This study presents updated Canadian estimates of the burden of hospitalizations attributable to influenza and RSV during the pre-pandemic periods spanning from 2010/2011 to 2013/2014, along with first-time estimates for influenza and RSV during the subsequent pre-pandemic periods from 2014/2015 to 2018/2019.
- This study introduces, for the first time, Canadian estimates of the burden of hospitalization attributable to seasonal respiratory viruses, including enterovirus, human metapneumovirus, human parainfluenza virus, and other related seasonal respiratory viruses. Endemic non-influenza/non-RSV respiratory viruses account for a sizable hospitalization burden in Canada and warrant continued monitoring.
- The rates of influenza- and RSV-associated hospitalizations in the seasons preceding the pandemic were generally increasing and have increased since the publication of the previous estimates in 2012. There are many external factors that could explain this increase, such as better case ascertainment due to more clinical testing and more sensitive testing methods; however, this change in testing and the refinement of the previous model may result in more accurate estimates going forward.
- Finally, our results demonstrate that the administrative hospitalization database, which is considered the most complete source for hospitalization data in Canada, under-represents the burden associated with seasonal influenza and RSV by 49% and 43%, respectively.

What are the key implications for public health interventions, practice, or policy?

- This analysis represents a milestone in the implementation of routine respiratory virus burden estimation into Canada’s respiratory virus surveillance program. Because administrative hospitalization databases under-represent the burden associated with seasonal influenza and RSV, producing annual model-based estimates of respiratory virus burden is essential for informing and evaluating public health policies, programs, and communications focused on mitigating the impacts of circulating respiratory viruses as well as maintaining core capacity planning in the health sector.
- Our results demonstrate that respiratory hospitalizations in the seasons preceding the pandemic were persisting and generally increasing. This trend reiterates the importance of public health interventions such as seasonal vaccination to lessen the pressures on Canada’s healthcare system, particularly during the winter months when viruses such as influenza and RSV are circulating at peak levels.
- With the introduction of new therapeutics (vaccines and monoclonal antibodies) for RSV and the expected increased usage in Canada, the updated RSV estimates (and the development of life stage–specific estimates) are especially important to monitor the effects of these new therapeutics in reducing RSV hospitalization burden.

## Data Availability

Not available.

## Code availability

Available upon request to the corresponding author.

## References

[CR1] Canadian Institute for Health Information. (2024). *Discharge Abstract Database (DAD) metadata*. Retrieved May 15, 2024, from https://www.cihi.ca/en/discharge-abstract-database-dad-metadata

[CR2] Canadian Institute for Health Information. (2006). *International statistical classification of diseases and related health problems, 10th revision, [Canada]: Tabular list* (Vol. 1).

[CR3] Chartrand, C., Tremblay, N., Renaud, C., & Papenburg, J. (2015). Diagnostic accuracy of rapid antigen detection tests for respiratory syncytial virus infection: Systematic review and meta-analysis. *Journal of Clinical Microbiology,**53*(12), 3738–3749. 10.1128/jcm.01816-15 26354816 10.1128/JCM.01816-15 PMC4652120

[CR4] Fleming, D. M., & Elliot, A. J. (2005). The impact of influenza on the health and health care utilisation of elderly people. *Vaccine,**23*, S1–S9. 10.1016/j.vaccine.2005.04.018 15908058 10.1016/j.vaccine.2005.04.018

[CR5] Gilca, R., De Serres, G., Skowronski, D., Boivin, G., & Buckeridge, D. L. (2009). The need for validation of statistical methods for estimating respiratory virus-attributable hospitalization. *American Journal of Epidemiology,**170*(7), 925–936. 10.1093/aje/kwp195 19679751 10.1093/aje/kwp195

[CR6] Goka, E. A., Vallely, P. J., Mutton, K. J., & Klapper, P. E. (2014). Mutations associated with severity of the pandemic influenza A(H1N1)pdm09 in humans: A systematic review and meta-analysis of epidemiological evidence. *Archives of Virology,**159*(12), 3167–3183. 10.1007/s00705-014-2179-z 25078388 10.1007/s00705-014-2179-z

[CR7] Iuliano, A. D., Roguski, K. M., Chang, H. H., Muscatello, D. J., Palekar, R., Tempia, S., Cohen, C., Gran, J. M., Schanzer, D., Cowling, B. J., Wu, P., Kyncl, J., Ang, L. W., Park, M., Redlberger-Fritz, M., Yu, H., Espenhain, L., Krishnan, A., Emukule, G., . . . Rodrigues, A. P. (2018). Estimates of global seasonal influenza-associated respiratory mortality: A modelling study. *Lancet*, *391*(10127), 1285–1300. 10.1016/s0140-6736(17)33293-2 10.1016/S0140-6736(17)33293-2 PMC5935243 29248255

[CR8] Li, X., Willem, L., Antillon, M., Bilcke, J., Jit, M., & Beutels, P. (2020). Health and economic burden of respiratory syncytial virus (RSV) disease and the cost-effectiveness of potential interventions against RSV among children under 5 years in 72 Gavi-eligible countries. *BMC Medicine*, *18*(1). 10.1186/s12916-020-01537-6 10.1186/s12916-020-01537-6 PMC7132892 32248817

[CR9] Mulpuru, S., Li, L., Ye, L., Hatchette, T., Andrew, M. K., Ambrose, A., Boivin, G., Bowie, W., Chit, A., Santos, G. D., ElSherif, M., Green, K., Haguinet, F., Halperin, S. A., Ibarguchi, B., Johnstone, J., Katz, K., Langley, J. M., LeBlanc, J., . . . Taylor, G. (2019). Effectiveness of influenza vaccination on hospitalizations and risk factors for severe outcomes in hospitalized patients with COPD. *Chest*, *155*(1), 69–78. 10.1016/j.chest.2018.10.044 10.1016/j.chest.2018.10.044 30616737

[CR10] Nair, H., Simões, E. A., Rudan, I., Gessner, B. D., Azziz-Baumgartner, E., Zhang, J. S. F., Feikin, D. R., Mackenzie, G. A., Moiïsi, J. C., Roca, A., Baggett, H. C., Zaman, S. M., Singleton, R. J., Lucero, M. G., Chandran, A., Gentile, A., Cohen, C., Krishnan, A., Bhutta, Z. A., . . . Campbell, H. (2013). Global and regional burden of hospital admissions for severe acute lower respiratory infections in young children in 2010: A systematic analysis. *Lancet*, *381*(9875), 1380–1390. 10.1016/s0140-6736(12)61901-1 10.1016/S0140-6736(12)61901-1 PMC3986472 23369797

[CR11] National Advisory Committee on Immunization. (2023). *National Advisory Committee on Immunization (NACI) statement: Seasonal influenza vaccine for 2023–2024*. Canada.ca. Retrieved February, 2023, from https://www.canada.ca/en/public-health/services/publications/vaccines-immunization/national-advisory-committee-immunization-statement-seasonal-influenza-vaccine-2023-2024.html

[CR12] Paget, J., Spreeuwenberg, P., Charu, V., Taylor, R. J., Iuliano, A. D., Bresee, J., Simonsen, L., & Viboud, C. (2019). Global mortality associated with seasonal influenza epidemics: New burden estimates and predictors from the GLaMOR Project. *Journal of Global Health*, *9*(2). 10.7189/jogh.09.020421 10.7189/jogh.09.020421 PMC6815659 31673337

[CR13] Patel, S. N., Gubbay, J. B., & Members of the Pandemic Influenza Laboratory Preparedness Network (PILPN). (2012). *Impact of pandemic influenza A (H1N1) on laboratory services* [Journal-article]. Retrieved January 2024, from https://nccid.ca/wp-content/uploads/sites/2/2015/03/NCCID_H1N1_impact_04.pdf

[CR14] Public Health Agency of Canada. (2023). *Seasonal influenza vaccination coverage in Canada, 2022–2023*. Canada.ca. Retrieved April 2024, from https://www.canada.ca/en/public-health/services/immunization-vaccines/vaccination-coverage/seasonal-influenza-survey-results-2022-2023/full-report.html

[CR15] Public Health Agency of Canada. (2024a). *Respiratory virus detections in Canada*. Canada.ca. Retrieved April 2024, from https://www.canada.ca/en/public-health/services/surveillance/respiratory-virus-detections-canada.html

[CR16] Public Health Agency of Canada. (2024b). Canadian Chronic Disease Surveillance System (CCDSS) [Data Tool]. Retrieved January 7, 2024, from https://health-infobase.canada.ca/ccdss/data-tool/

[CR17] Public Health Agency of Canada. (2025). *Canadian respiratory virus surveillance report*. Canada.ca. Retrieved October, 2024, from https://health-infobase.canada.ca/respiratory-virus-surveillance/

[CR18] Schanzer, D. L., McGeer, A., & Morris, K. (2012). Statistical estimates of respiratory admissions attributable to seasonal and pandemic influenza for Canada. *Influenza and Other Respiratory Viruses,**7*(5), 799–808. 10.1111/irv.12011 23122189 10.1111/irv.12011 PMC3796862

[CR19] Schanzer, D. L., Saboui, M., Lee, L., Nwosu, A., & Bancej, C. (2017). Burden of influenza, respiratory syncytial virus, and other respiratory viruses and the completeness of respiratory viral identification among respiratory inpatients, Canada, 2003–2014. *Influenza and Other Respiratory Viruses,**12*(1), 113–121. 10.1111/irv.12497 29243369 10.1111/irv.12497 PMC5818333

[CR20] Schanzer, D. L., Sevenhuysen, C., Winchester, B., & Mersereau, T. (2013). Estimating influenza deaths in Canada, 1992–2009. *PLoS ONE,**8*(11), e80481. 10.1371/journal.pone.0080481 24312225 10.1371/journal.pone.0080481 PMC3842334

[CR21] Shi, T., Denouel, A., Tietjen, A. K., Campbell, I., Moran, E., Li, X., Campbell, H., Demont, C., Nyawanda, B. O., Chu, H. Y., Stoszek, S. K., Krishnan, A., Openshaw, P., Falsey, A. R., Nair, H., & Investigators, R. (2019). Global disease burden estimates of respiratory syncytial virus–associated acute respiratory infection in older adults in 2015: A systematic review and meta-analysis. *The Journal of Infectious Diseases,**222*(Suppl 7), S577–S583. 10.1093/infdis/jiz059 10.1093/infdis/jiz059 30880339

[CR22] Statistics Canada. (2016). *Census in Brief: A portrait of the population aged 85 and older in 2016 in Canada, Census year 2016*. Retrieved April 2024, from https://www12.statcan.gc.ca/census-recensement/2016/as-sa/98-200-x/2016004/98-200-x2016004-eng.cfm

[CR23] Statistics Canada. (2024). *Population estimates on July 1, by age and gender*. Retrieved January 9, 2023, from https://www150.statcan.gc.ca/t1/tbl1/en/tv.action?pid=1710000501

[CR24] Thompson, W. W., Shay, D. K., Weintraub, E., Brammer, L., Bridges, C. B., Cox, N. J., & Fukuda, K. (2004). Influenza-associated hospitalizations in the United States. *JAMA,**292*(11), 1333. 10.1001/jama.292.11.1333 15367555 10.1001/jama.292.11.1333

[CR25] Thompson, W. W., Shay, D. K., Weintraub, E., Brammer, L., Cox, N., Anderson, L. J., & Fukuda, K. (2003). Mortality associated with influenza and respiratory syncytial virus in the United States. *JAMA,**289*(2), 179. 10.1001/jama.289.2.179 12517228 10.1001/jama.289.2.179

[CR26] Troeger, C. E., Blacker, B. F., Khalil, I. A., Zimsen, S. R. M., Albertson, S. B., Abate, D., Abdela, J., Adhikari, T. B., Aghayan, S. A., Agrawal, S., Ahmadi, A., Aichour, A. N., Aichour, I., Aichour, M. T. E., Al-Eyadhy, A., Al-Raddadi, R. M., Alahdab, F., Alene, K. A., Aljunid, S. M., . . . Defo, B. K. (2019). Mortality, morbidity, and hospitalisations due to influenza lower respiratory tract infections, 2017: An analysis for the Global Burden of Disease Study 2017. *The Lancet. Respiratory Medicine*, *7*(1), 69–89. 10.1016/s2213-2600(18)30496-x 10.1016/S2213-2600(18)30496-X PMC6302221 30553848

[CR27] World Health Organization. (2015). *A manual for estimating disease burden associated with seasonal influenza*. Retrieved April 2024, from https://www.who.int/publications/i/item/9789241549301

[CR28] Wong, C. M., Yang, L., Chan, K. P., Leung, G. M., Chan, K. H., Guan, Y., Lam, T. H., Hedley, A. J., & Peiris, J. S. M. (2006). Influenza-associated hospitalization in a subtropical city. *PLoS Medicine,**3*(4), e121. 10.1371/journal.pmed.0030121 10.1371/journal.pmed.0030121 PMC1391978 16515368

[CR29] Yang, L., Chiu, S. S., Chan, K., Chan, K., Wong, W. H., Peiris, J. S. M., & Wong, C. (2011). Validation of statistical models for estimating hospitalization associated with influenza and other respiratory viruses. *PLoS ONE,**6*(3), e17882. 10.1371/journal.pone.0017882 21412433 10.1371/journal.pone.0017882 PMC3055891

